# Supplementary material for: Human iPSC Modeling of Genetic Febrile Seizure Reveals Aberrant Molecular and Physiological Features Underlying an Impaired Neuronal Activity
Source: Biomedicines. 2022 May 5;10(5):1075. doi: 10.3390/biomedicines10051075 (PMC9138645; doi:10.3390/biomedicines10051075)
Supplement: Supplementary file 1 [file biomedicines-10-01075-s001.zip › biomedicines-1702942-supplementary.pdf]

Supplementary Materials

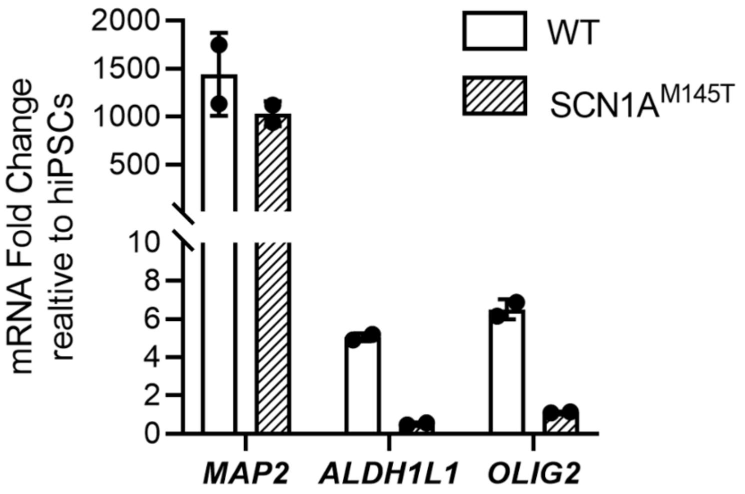

Figure S1. Differentiated idNs presented low levels of astrocyte marker *ALDH1L1* and oligodendrocyte marker *OLIG2*, compared to the expression of neuronal marker *MAP2*. *GAPDH* was used as control. Data are presented as mean ± SEM of two biological replicates (black dots).

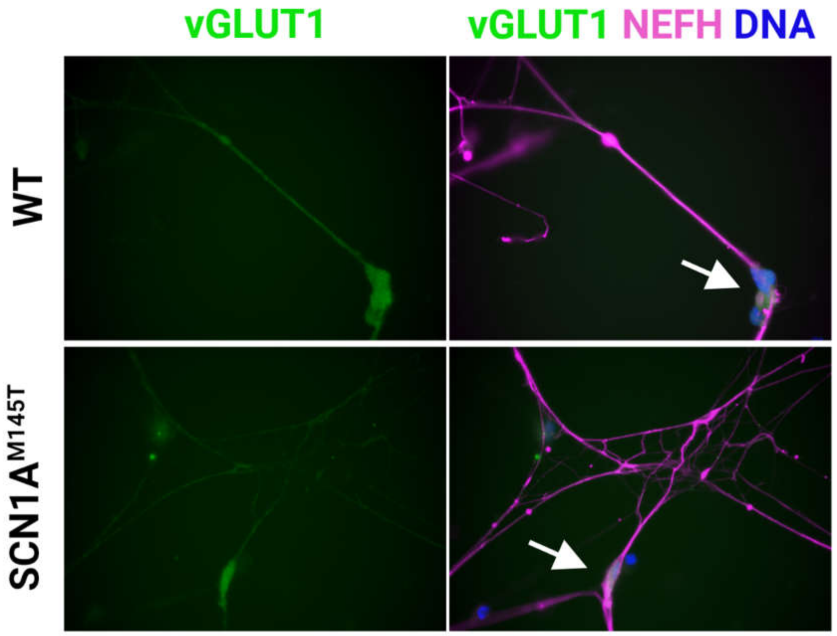

Figure S2. Immunofluorescence analysis of vesicular glutamate transporter vGLUT1 expression compared to neuronal marker NEFH in idNs of WT (upper images) and SCN1A^{M145T} (lower images) subjects. White arrows indicate idNs positive for vGLUT1.

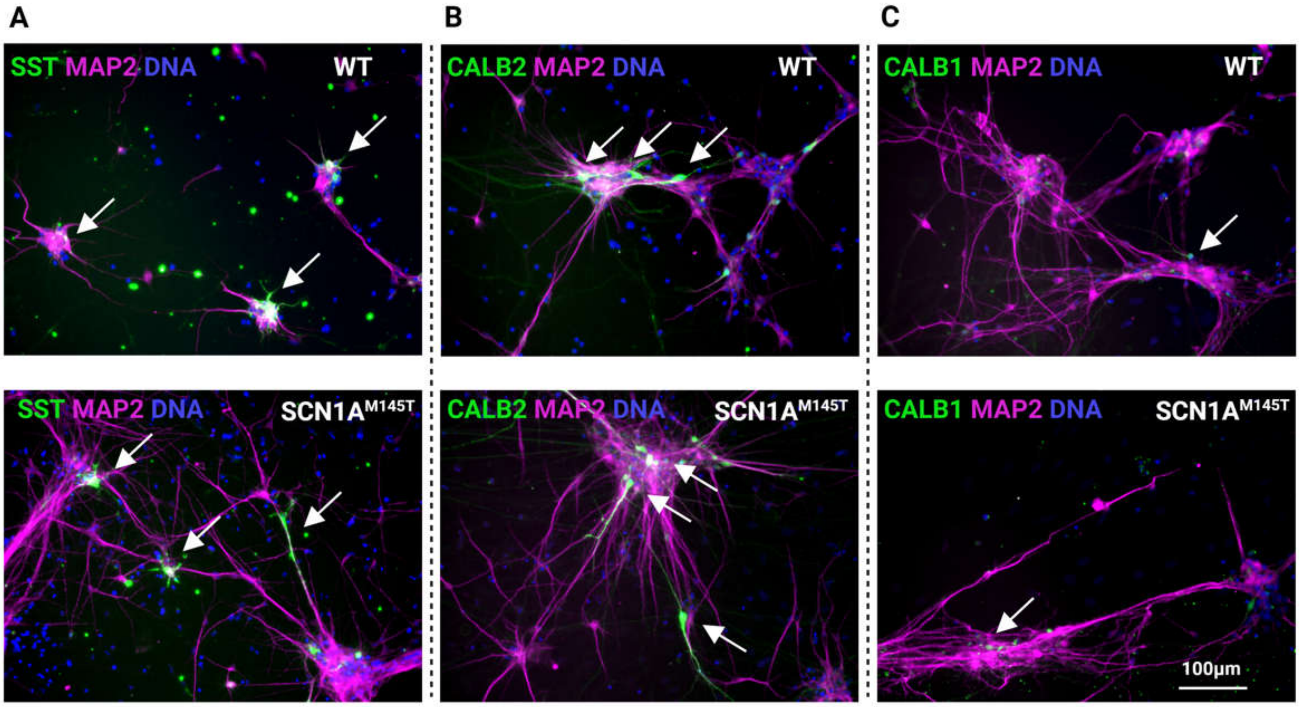

Figure S3. Immunofluorescence analysis of idNs showing the interneuronal subtype markers distribution compared to MAP2 expression: (**A**), somatostatin (SST) (**B**), calretinin (CALB2), and (**C**), calbindin (CALB1). Nuclei are stained in blue with DAPI. For each marker tested, WT-idNs are shown in the upper panels, while SCN1A^{M145T} idNs are shown in the lower panels. Arrows indicate neurons expressing the specific interneuronal makers.

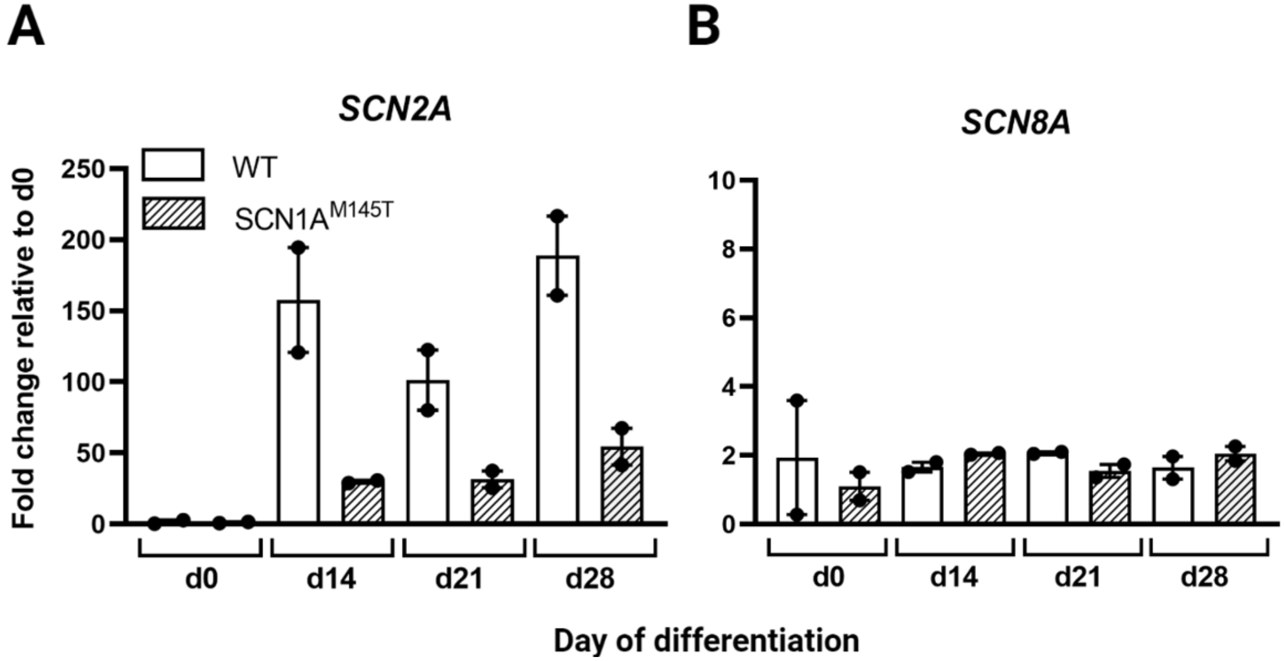

Figure S4. Quantitative RT-PCR analysis of CNS VSVGs genes in WT-idNs and SCN1A^{M145T}-idNs at day of differentiation 0 (NSCs), d14, d21, and d28. (**A**), *SCN2A* results expressed in both, control and diseased idNs, but shows a significantly higher expression in the WT cells. (**B**), *SCN8A*, the adult isoform, is the VSVGs with the lowest expression among both WT and SCN1A^{M145T} groups during all time points analyzed. Data are presented as mean±SEM of two biological replicates (black dots).

Table S1. List of primers used in qRT-PCR experiments.

Gene Name	Forward Primer	Reverse Primer
GAPDH	TCCTCTGACTTCAACAGCGA	GGGTCTTACTCCTTGGAGGC
MAP2	CCACCTGAGATTAAGGATCA	GGCTTACTTTGCTTCTCTGA
NEFM	TCCTCAACGTCAAGATGGCT	GTGTTGGACCTTAAGCTTGGG
NEFL	AGACCCTGGAAATCGAAGCA	TCACGTTGAGGAGGTCTTGG
SYP	CAAGGGGCTGTCAGATGTGA	CCTGTCTCCTTAAACACGAACC
PSD95	CGTTCGCCTCTATGTCATGC	TCCAATCTGCAACCTCCCAT
GAD2	CTCATTGCCTTCACGTCTGA	GCTGTCTGTTCCAATCCCTAA
vGLUT2	GACCTACCCAGCATGTCATG	ACCAGACCATTCCAAAGCTTC
SCN1A	GTGTGGTTTCCTTGGTTGGT	GTCCATGGAAACGTGGAAAG
SCN2A	ATCAGGCCACATTGGAAGAG	GATGCTACTGAAGAACTCTCTG AAAA
SCN3A	GCCAAACCATGTGCCTTATT	CCCTTTTGCATTCTTCCTACTG
SCN8A	GGGGAACCTTCGAAACAAGT	GCATCAGAACTGTTCCCACA
KCC2	GCCACGCTTTCGATATTACC	GCATGGCTACCAGTGCATAA
NKCC1	TGACTTGAGAGAAGGTGCACAG	TGTTTGGCTTCATACGACCA
